# High concentrations of illicit stimulants and cutting agents cause false positives on fentanyl test strips

**DOI:** 10.1186/s12954-021-00478-4

**Published:** 2021-03-09

**Authors:** Tracy-Lynn E. Lockwood, Alexandra Vervoordt, Marya Lieberman

**Affiliations:** Department of Chemistry and Biochemistry, University of Notre Dame, 251 Stepan Hall of Chemistry, Notre Dame, IN 46556 USA

**Keywords:** Fentanyl test strip (FTS), Opioid, Harm reduction, Drug testing, False positives, Fentanyl, Stimulant

## Abstract

**Background:**

The opioid epidemic has caused an increase in overdose deaths which can be attributed to fentanyl combined with various illicit substances. Drug checking programs have been started by many harm reduction groups to provide tools for users to determine the composition of their street drugs. Immunoassay fentanyl test strips (FTS) allow users to test drugs for fentanyl by either filling a baggie or cooker with water to dissolve the sample and test. The antibody used in FTS is very selective for fentanyl at high dilutions, a characteristic of the traditional use of urine testing. These street sample preparation methods can lead to mg/mL concentrations of several potential interferents. We tested whether these concentrated samples could cause false positive results on a FTS.

**Methods:**

20 ng/mL Rapid Response FTS were obtained from BTNX Inc. and tested against 4 different pharmaceuticals (diphenhydramine, alprazolam, gabapentin, and naloxone buprenorphine) and 3 illicit stimulants [cocaine HCl, methamphetamine, and 3,4-methylenedioxymethamphetamine (MDMA)] in concentrations from 20 to 0.2 mg/mL. The FTS testing pad is divided into 2 sections: the control area and the test area. Control and test area signal intensities were quantified by ImageJ from photographs of the test strips and compared to a threshold set by fentanyl at the FTS limit of detection.

**Results:**

False positive results indicating the presence of fentanyl were obtained from samples of methamphetamine, MDMA, and diphenhydramine at concentrations at or above 1 mg/mL. Diphenhydramine is a common cutting agent in heroin. The street sample preparation protocols for FTS use suggested by many online resources would produce such concentrations of these materials. Street samples need to be diluted more significantly to avoid interference from potential cutting agents and stimulants.

**Conclusions:**

Fentanyl test strips are commercially available, successful at detecting fentanyl to the specified limit of detection and can be a valuable tool for harm reduction efforts. Users should be aware that when drugs and adulterants are in high concentrations, FTS can give a false positive result.

**Supplementary Information:**

The online version contains supplementary material available at 10.1186/s12954-021-00478-4.

## Background

The opioid overdose epidemic is a national emergency in America [1]. Since 1999, more than 750,000 deaths have been attributed to an opioid-induced overdose [2]. In the following two decades, four distinct waves of opioid overdose deaths have occurred. In the first wave, increased access to prescription opioids was the primary cause of new overdose related deaths. The second wave, beginning in 2010, was characterized by a rapid increase in deaths due to heroin overdoses. The third wave began in 2013, at which point Fentanyl became a leading cause of overdose deaths [3]. Now, in 2020, we are in what is being called the 4th wave of the opioid crisis—stimulants such as cocaine and methamphetamine or depressants like benzodiazepines combined with opioids [4–7].

Current research suggests that people who use illicit drugs often do not know whether fentanyl is present in what they are about to consume [8]. Fentanyl first rose to prominence in the 1960s due to its effectiveness as a painkiller. It has since become a popular—and dangerous—substance that is directly associated with the 3rd and 4th wave of the epidemic. Fentanyl is 75–100 times more potent then morphine [9]. Although there was a 4.1% decrease in opioid deaths in 2018 compared to 2017, the rate of drug overdose involving fentanyl, fentanyl analogs, and tramadol increased by 10% [4]. In order to respond to this epidemic, harm reduction practices are being explored by public health organizations [10].

In the context of this paper, “harm reduction” is defined as programs and policies that aim to reduce the dangers associated with drug use. Harm reduction, therefore, exists as a preventative measure focusing on reducing drug-related harm [11]. Harm reduction programs started at the height of the AIDS epidemic in the early 1990s primarily serving as syringe exchange sites to limit transfer of the disease among IV drug users [12]. Many of these programs have broadened their services to not only include syringe exchange access, but access to counseling and support services, and most recently drug checking initiatives [13, 14].

Drug checking abilities have become desired services in the harm reduction world to inform the user of the composition (and potential contaminants) present in their drugs [13]. Examples of drug checking methods include liquid reagents, Fourier-transform infrared spectroscopy (FTIR), Raman Spectroscopy, High-Pressure Mass Spectrometry (HPMS), Thin-layer chromatography (TLC), Immunoassay Test strips, high-performance liquid chromatography (HPLC), Gas chromatography–mass spectrometry (GC–MS), Liquid chromatography–mass spectrometry (LC–MS), among others [13]. As analytical techniques such as FTIR and HPMS instruments become more portable and cost friendly, many harm reduction programs are purchasing these devices to provide community aid. However, liquid reagent kits and immunoassay test strips remain at the forefront of harm reduction measures due to accessibility and cost. The immunoassay test strips are of particular use in detecting fentanyl and fentanyl analogues because of the reliable detection at low concentrations and complex matrices that are often missed by spectroscopy methods [15, 16].

In order to help prevent overdoses, lateral flow immunoassay test strips originally designed for monitoring traces of fentanyl and its analogs in urine are being explored as a drug checking technology in harm reduction contexts [17–20]. One commonly used fentanyl test strip or “FTS” (BTNX Inc., Markham, ON, Canada) is a lateral flow chromatographic immunoassay for the qualitative detection of fentanyl in urine at the cutoff concentration of 20 ng/mL. A positive result on this test strip gives one line, a negative result gives two lines, and an invalid test gives either no line or no control line [21]. The “off label” use of the FTS in a harm reduction context involves preparation of a solution of the drug to be checked. For example, the residue in a cooker or baggie may be dissolved in a little water and then tested with the FTS. BTNX Inc. provides information about specificity of their test strip response, but for fentanyl 20 ng/mL FTS, the only drugs tested were fentanyl (detected at 20 ng/mL in urine) and norfentanyl (detected at 375 ng/mL in urine). In addition, a suite of pharmaceuticals were found to be non-interfering at levels of 100 ug/mL in a urine matrix [21, 22]. We have found that common stimulants and cutting agents that are often present in illicit drugs can create false positives. The problem arises from the cross-reactivity of the antibody for these other substances [23]. Although the affinity of the antibody for these substances is much lower than for fentanyl, if they are present at sufficiently high concentrations, they can cause a false positive result [24, 25]. As we consider the 4th wave of the pandemic, it can be expected that drug users will need to test stimulants to see if they contain fentanyl.

We tested BTNX. Inc. 20 ng/mL immunoassay fentanyl test strips against 4 pharmaceuticals (diphenhydramine, alprazolam, Gabapentin, and naloxone buprenorphine) and 3 illicit stimulants (cocaine HCl, methamphetamine, and 3,4-methylenedioxymethamphetamine (MDMA)) to determine the prevalence of false positives at concentrations from 20 to 0.2 mg/mL. These substances were selected based on advice from harm reduction groups. Further, we were able to determine a suggested sample dilution and time for reading and interpretation of the results that will detect dangerous levels of fentanyl with less risk of false positives.

## Methods

### Fentanyl test strips

Rapid Response Fentanyl Test Strips (FTS) were procured from BTNX Inc. (20 ng/mL, Lots D808009 and DOA903194, Markham, ON, Canada). Fentanyl test strips were kept in their sealed packaging until immediately before use. Each strip was dipped with the blue wavy line side, arrows pointing down, into an aliquot of solution for approximately 12–15 s (until solution reached testing pad). Strips were then placed on an absorbent, flat surface for 5 min, following manufacturer’s instructions. The FTS testing pad is divided into 2 sections: the control area and the test area. The presence of a pink band in the control area is an indication that the test strip preformed properly. The presence of a pink band in the test area is an indication that the analyte (in this case, fentanyl) is not present, while the absence of a pink band in the test area indicated detection of the analyte. Photographs were taken of FTS using an iPhone 11 under ambient laboratory lighting.

### Test substances

Diphenhydramine tablets and capsules were purchased from a local grocery store (Top Care Brand, 25 mg). Analytical grade fentanyl standard was purchased from Sigma Aldrich (Cerilliant 1 mg/mL in 1 mL Methanol, Lot FE12281801). Alprazolam tablets, Gabapentin capsules, and Naloxone Buprenorphine tablets were obtained from the Marion County Deputy Coroner’s Office as artifacts from accidental overdose deaths. Sample identities were confirmed using pharmaceutical pill databases and liquid chromatography–mass spectrometry (LC–MS). Cocaine HCl, methamphetamine, and 3,4-methylenedioxymethamphetamine (MDMA) were obtained from the Berrien County Forensic Laboratory as independent drug seizures. Street sample identity and purity was confirmed using Fourier-transform infrared spectroscopy (FTIR) and gas chromatography–mass spectrometry (GC–MS). These substances were selected based on reports from drug checkers at the Chicago Recovery Alliance and the perceived false positives on FTS during their analysis.

### Fentanyl analysis

One vial of 1 mg/mL fentanyl standard in methanol was utilized for fentanyl analysis. The methanol in the standard was evaporated gently on a hot plate. Deionized water (DI) water was used to dilute the fentanyl volumetrically from 1 to 0.005 mg/mL. The solution was then serially diluted to 5 ng/mL to determine fentanyl test strip limit of detection. One FTS was used at each dilution until 125 ng/mL, 100 ng/mL, 83 ng/mL, 63 ng/mL, 50 ng/mL, 25 ng/mL, and 5 ng/mL where 5 strips were used at each dilution.

### Interference study

Twenty milligrams of each interferent was weighed out on an analytical balance and put into independently labeled vials. Solid samples were initially dissolved in 1 mL of DI water for a concentration of 20 mg/mL. The solution was then further diluted in 2 mL (10 mg/mL), 3 mL (6.7 mg/mL), 5 mL (4 mg/mL), 8 mL (2.5 mg/mL), 10 mL (2 mg/mL), 20 mL (1 mg/mL), 30 mL (0.67 mg/mL), 40 mL (0.50 mg/mL), 50 mL (0.40 mg/mL), 60 mL (0.33 mg/mL), 70 mL (0.29 mg/mL), 80 mL (0.25 mg/mL), 90 mL (0.22 mg/mL), and 100 mL (0.20 mg/mL) with one FTS used at each dilution. The FTS was placed in the solution for 12–15 s following the procedure above. Photographs were taken of FTS five minutes after dipping in solution per manufacturer’s instructions.

### Time study

A FTS was dipped in DI water for 12 s and placed on a flat surface. Photographs were taken every 5 s of the test strip for the first 2 min (120 s) and every 10 s until 7 min (420 s). To analyze the fentanyl standard development time, a FTS was dipped in 1 mL of 1 mg/mL fentanyl standard for 12 s until the solution reached the testing pad and placed on a flat surface. Photographs were taken following the same procedure used for water.

### Image analysis

FTS were analyzed using NIH ImageJ software [26] for the interference and time studies. Images were converted to 32-bit black and white with contrast corrected to [50.293, 205.063]. The ImageJ gel analysis tool was used for band analysis; the pixel values were plotted and integrated to get peak area counts.

## Results

### Interference study

**Fig. 1:**
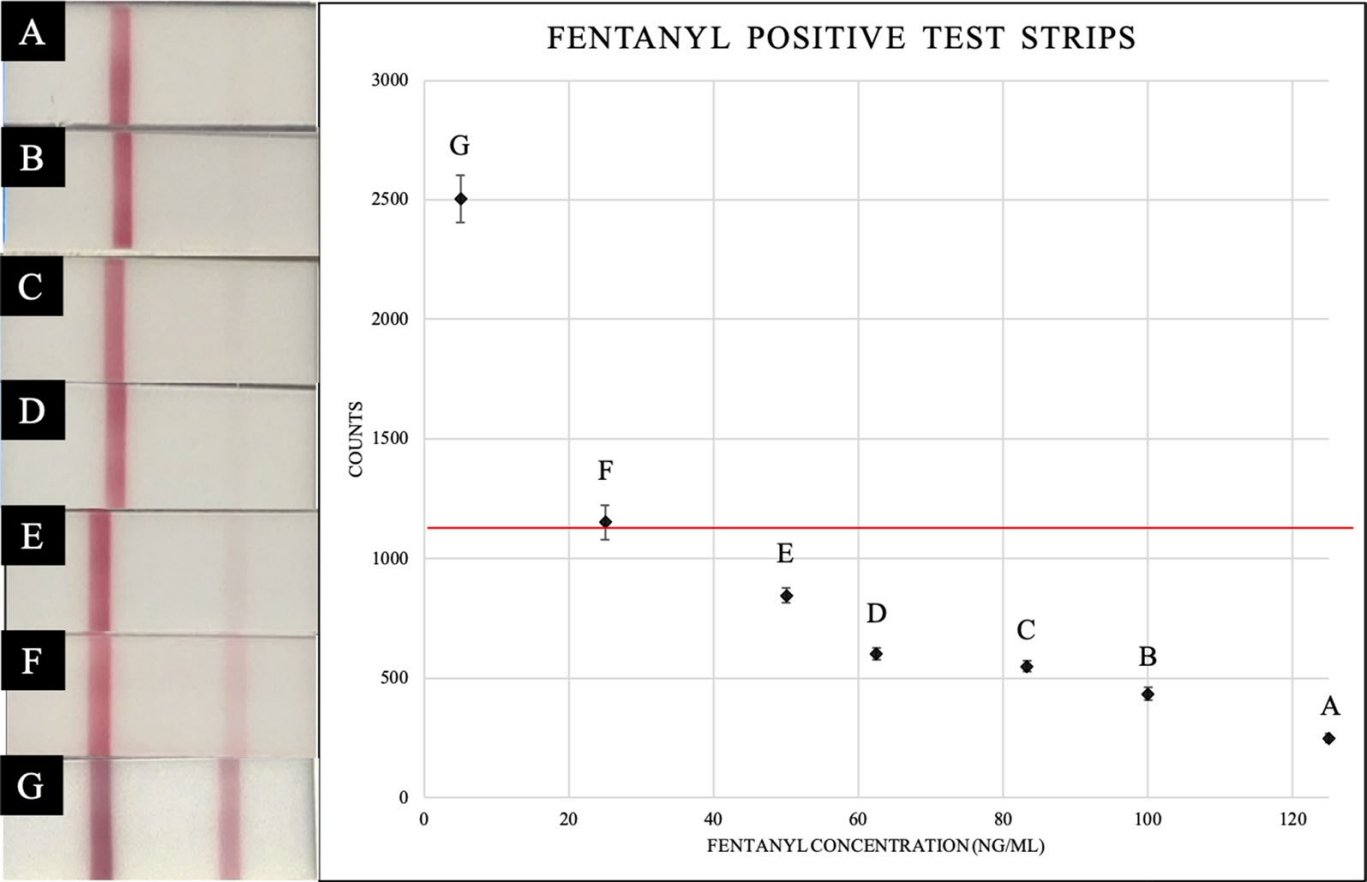
FTS with fentanyl standard: FTS ran with fentanyl standard at 125 ng/mL (**a**), 100 ng/mL (**b**), 83 ng/mL (**c**), 63 ng/mL (**d**), 50 ng/mL (**e**), 25 ng/mL (**f**), and 5 ng/mL (**g**). Fentanyl threshold line determined to be at 25 ng/mL correlating to 1152 counts (SD = 72)

Positive fentanyl solutions were prepared at 1 mg/mL and diluted down to 5 ng/mL. The FTS limit of detection (LOD) was determined by eye to be 25 ng/mL due to the presence of a dark testing band (Fig. 1, Left). Quantification of the testing bands correlates 25 ng/mL to 1152 counts (*n* = 5, SD = 72) (Fig. 1, Right) and is consistent with the FTS packaging stating a 20 ng/mL detection limit. The LOD value will be referenced as the fentanyl threshold line.

**Fig. 2:**
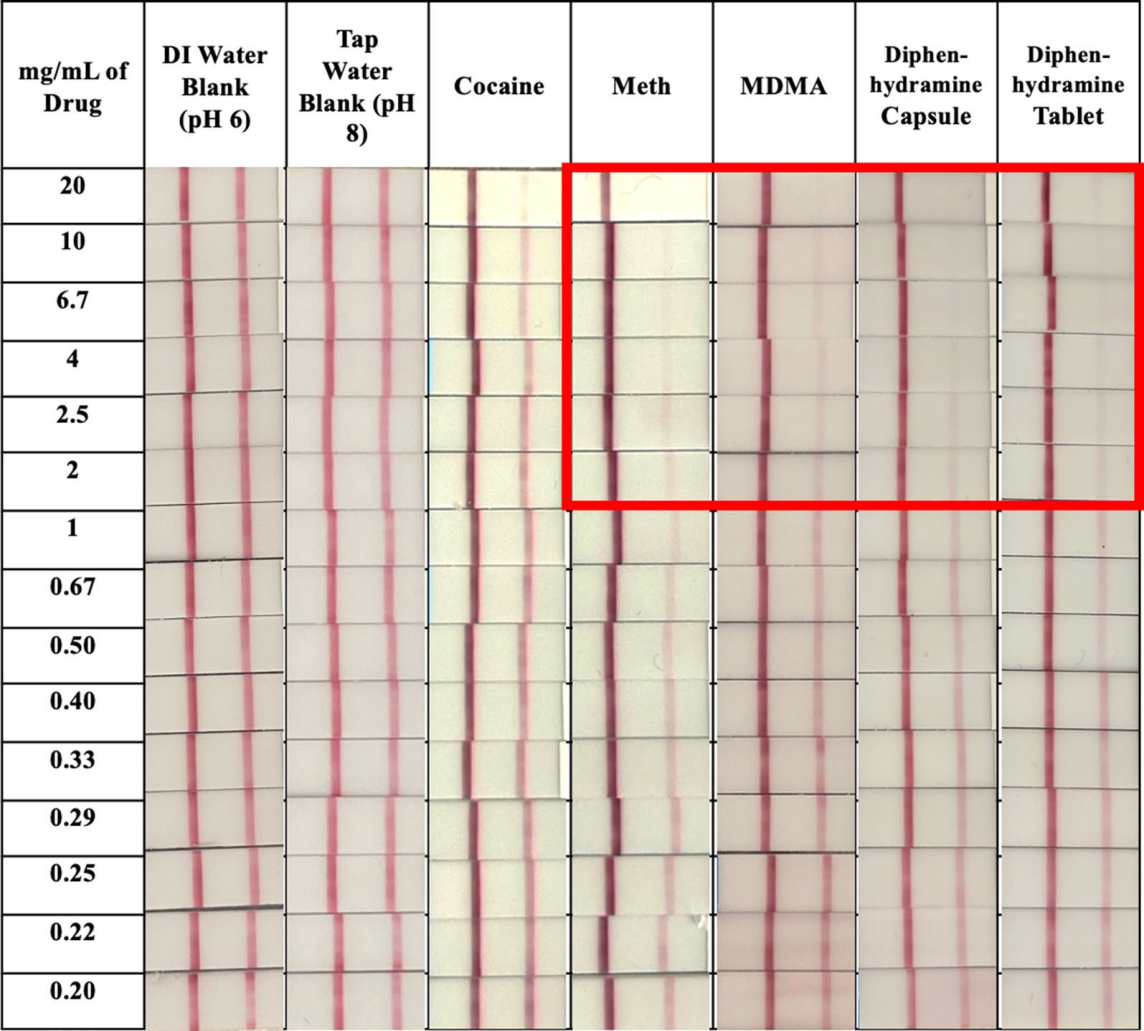
Fentanyl test strip images of interference compounds: FTS were ran with water, cocaine, methamphetamine, MDMA, diphenhydramine capsules, and diphenhydramine tablets and were photographed after 5 min. The testing bar (right side of testing pad) for moderately concentrated samples (approximately > 2 mg/mL) of methamphetamine, MDMA, diphenhydramine capsules, diphenhydramine tablets did not appear indicating false positives

**Fig. 3:**
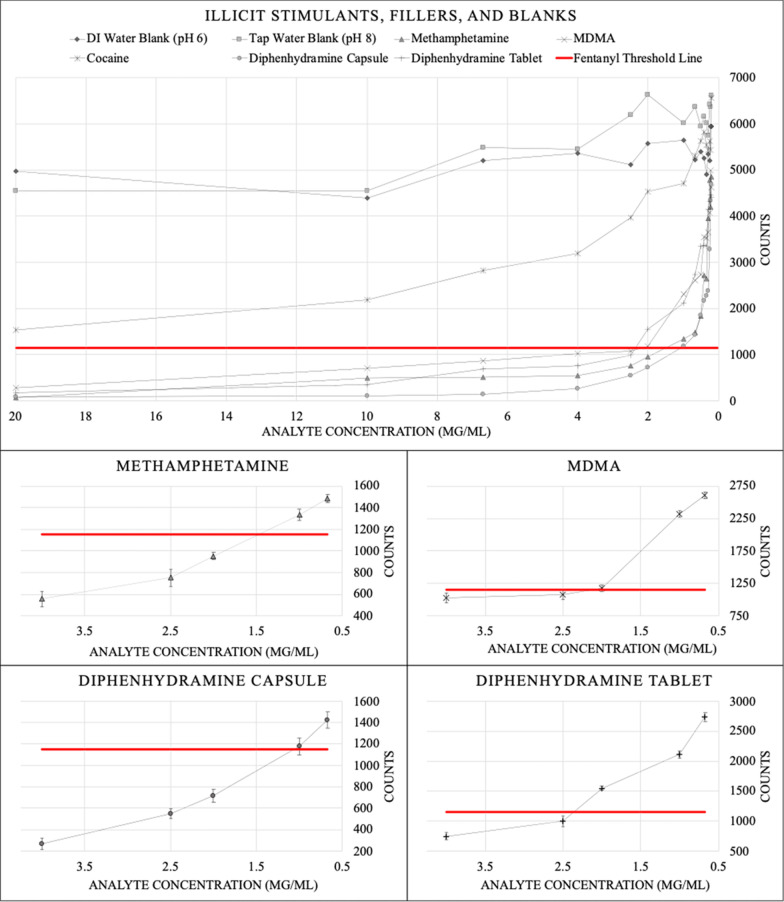
Illicit stimulants, fillers, and blanks: samples with intensities below the red line are likely to be mistaken for fentanyl. Sub-graphs for each substance that crosses the fentanyl threshold line are included with error bars for multiple measurements

The FTS were assessed to determine prevalence of false positives when tested with stimulants such as cocaine, methamphetamine, and MDMA as well as over-the-counter medicines such as alprazolam, gabapentin, naloxone buprenorphine, and diphenhydramine. Solutions were prepared at 20 mg/mL in DI water and diluted down to 0.2 mg/mL. By eye, FTS with cocaine, alprazolam, gabapentin, and naloxone buprenorphine were negative even at the highest concentration, with the appearance of dark bands in both the control and test regions (Additional file 1). However, the FTS testing band with methamphetamine, MDMA, and both diphenhydramine capsules and tablets did not appear when the analytes were in moderate concentrations (> 2 mg/mL). These samples could be read as false positives (Fig. 2). Figure 3 shows the integrated intensities of the test bands; the error bars are the standard deviation from replicate measurements (sub-graphs shown for each substance close to the fentanyl detection limit). The integrated intensities of test bands from solutions of cocaine, DI water, and tap water (which should all be negative for fentanyl) were well above the fentanyl threshold. However, the integrated intensities of the test bands from moderately concentrated samples of methamphetamine, MDMA, diphenhydramine capsules, and diphenhydramine tablets were below the fentanyl threshold, consistent with the visual false positive results. The critical concentration level for diphenhydramine capsules was 1 mg/mL, for diphenhydramine tablets 2.5 mg/mL, methamphetamine was 1.5 mg/mL, and MDMA was 2 mg/mL. At or above these concentrations, the FTS is likely to produce a false positive result.

While both the diphenhydramine tablets and capsules contain 25 mg of diphenhydramine HCl active ingredient, these are over-the-counter formulations. The diphenhydramine capsule’s average weight is 240 mg (*n* = 3, SD = 3), whereas the tablet’s average weight is 250 mg (*n* = 3, SD = 4). In this case, the tablets contain more cutting agents so the 20 mg of solid used for the test would have less diphenhydramine for the tablet measurement than the capsule and is supported by the lower false positive critical concentration.

FTS were ran with diphenhydramine tablets and capsules in three different temperature conditions (4 °C, 25 °C (Room Temperature), and 40 °C) at three different concentrations (4 mg/mL, 2 mg/mL, and 1 mg/mL). FTS were ran and allowed 5 min to process in these temperature conditions. These concentrations were selected to cover the critical concentration of false positives for both diphenhydramine capsules and tablets. In the room temperature and warm conditions with the diphenhydramine tablet, the FTS behaved as expected with a false positive result at 4 mg/mL and negative results for 2 mg/mL and 1 mg/mL (critical concentration of 2.5 mg/mL). In the room temperature and warm conditions with the diphenhydramine capsule, the FTS also behaved as expected with false positive results at 4 mg/mL and 2 mg/mL with a negative result for 1 mg/mL (critical concentration of 1 mg/mL). In the cold condition, all FTS gave a false positive result (only appearance of control band). The FTS test band does not react properly at temperatures at or below 4 °C. Images were taken 5 min after running and can be found in Additional file 1.

### Time study

**Fig. 4:**
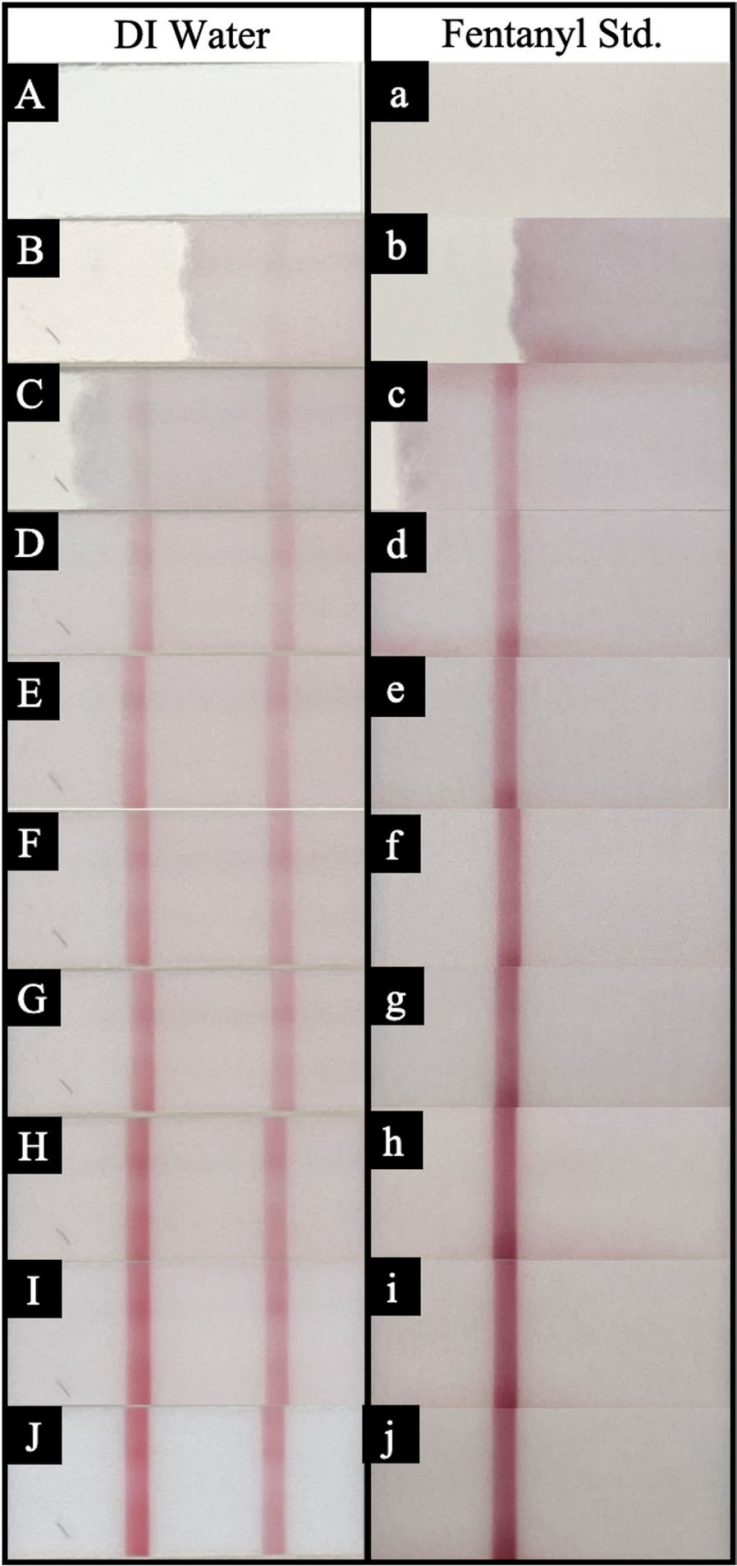
Color development of control and test bars for water and fentanyl: **A**–**J**: Water, Panels **a**–**j**: 1 mg/mL Fentanyl standard. **A**/**a**: testing pad prior to solution saturation; Panel **B**/**b**, 10 s after solution reached testing pad; Panel **C**/**c**, 20 s; Panel **D**/**d**, 30 s, Panel **E**/**e**, 40 s; Panel **F**/**f**, 50 s; Panel **G**/**g**, 60 s; Panel **H**/**h**, 120 s; Panel **I**/**i**, 240 s, Panel **J**/**j**, 420 s

**Fig. 5:**
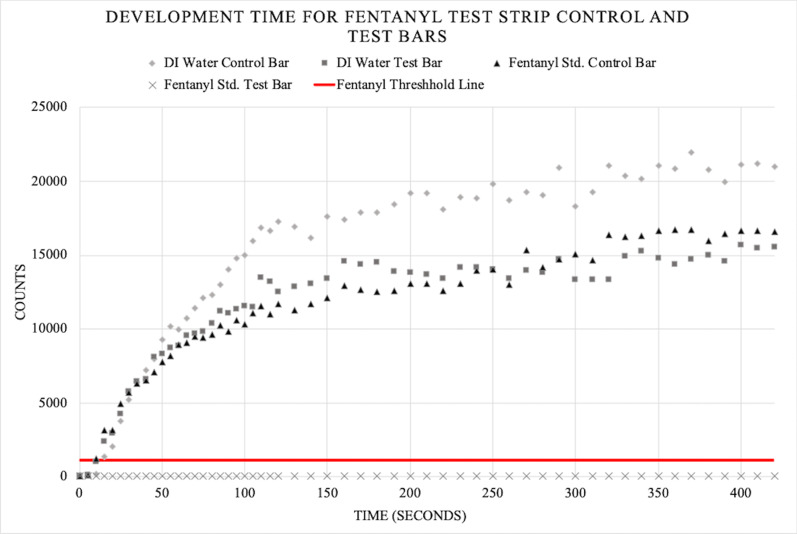
Development time for fentanyl test strip control and test bars: FTS were tested in DI water and 1 mg/mL fentanyl standard. Photographs were taken every 5 s of test strips up to 420 s. The integrated intensities of the control and test bars were analyzed using ImageJ

FTS were analyzed to determine how long a user should wait prior to reading the results. Panels A–J in Fig. 4 show images of a water control FTS. Panels a–j in Fig. 4 show the FTS images for a 1 mg/mL fentanyl standard solution. These photographs were quantitatively analyzed using ImageJ and are plotted in Fig. 5. The BTNX Inc. FTS product insert states to read the FTS between 5 and 10 min after sampling [21, 22].

There is significant color change of the water control, water test, and fentanyl control bars over the first 120 s after running. Fentanyl test bar values never leave the x-axis due to the bar never developing in a positive fentanyl sample. The water control, water test, and fentanyl control bars develop at a rate of 143, 104, and 97 counts per second, respectively, over the first 120 s. From 120 s to the final measurement at 420 s, the water control, water test, and fentanyl control bars develop at a rate of 12, 10, and 16 counts per second indicating approximately a 10 × color development rate over the first 2 min (120 s). After this point, both the testing and control bands become saturated and the color development rate levels out. From this data, a user should wait 2 min (120 s) at minimum when interpreting FTS.

The fentanyl threshold line is included in Fig. 5 to also show how quickly the test bars develop in comparison to the LOD of fentanyl detection. By approximately 15 s, the user should be able to determine if a test band will appear by eye; however, the manufacturer’s instructions should be followed when possible.

## Discussion

In a harm reduction setting, a FTS might be used to test the drug residue in a cooker or baggie for fentanyl before use of the drug. Our results show that the concentrations of diphenhydramine, methamphetamine, and MDMA commonly found in street drugs are at levels that could generate false positives on the FTS. Many cookers and small baggies hold about 0.75–1 mL of water. If we assume there is 5 mg of methamphetamine in the container that is diluted with 1 mL of water, the concentration of methamphetamine will be 5 mg/mL and would trigger a false positive on the FTS. If the residue were dissolved with 10 mL of water, the methamphetamine concentration would be 0.5 mg/mL and would render a true negative on the FTS. If the drug residue instead consisted of 95% methamphetamine and 5% fentanyl, the 10 mL dilution would ensure that the methamphetamine concentration would not interfere with the FTS while the true positive result would come from the fentanyl present in the sample. As practical guidance for harm reduction groups, a dilution with at least 50 mL of water will provide a good margin of error for accurate detection of fentanyl in cooker or powder residues while avoiding false positives from other drugs. Over dilution is not a likely problem; the FTS is sensitive enough that if there was just 0.5 mg of fentanyl residue in a cooker and it is dissolved in a 10-L bucket of water (50 µg/L or 50 ng/mL), the FTS will still detect the fentanyl present.

## Conclusion

Drug checking initiatives have become an increasingly popular tool in harm reduction programs allowing users to test their supply prior to use. One widely used product is the BTNX Inc. Rapid Response Fentanyl Test Strip (FTS) due to its quick and easy analysis of fentanyl and multiple fentanyl analogs. The FTS is commercially available, successful at detecting fentanyl to the specified limit of detection and can be a valuable tool for harm reduction efforts. Users should be aware that when potential drug adulterants are in high concentrations, the FTS can give a false positive result. Samples for drug checking should be significantly diluted to avoid false positives from diphenhydramine, methamphetamine, and MDMA.

## Supplementary Information

**Additional file 1**. Supplementary Material.

## Data Availability

All data generated or analyzed during this study are included in this published article (and its supplementary information files).

## Abbreviations

DI water: Deionized water
FTIR: Fourier-transform infrared spectroscopy
FTS: Fentanyl test strip
GC–MS: Gas chromatography–mass spectrometry
HPLC: High-performance liquid chromatography
HPMS: High-pressure mass spectrometry
LC–MS: Liquid chromatography–mass spectrometry
LOD: Limit of detection
MDMA: 3,4-Methylenedioxymethamphetamine
TLC: Thin-layer chromatography
