# Supplementary material for: High concentrations of illicit stimulants and cutting agents cause false positives on fentanyl test strips
Source: Harm Reduct J. 2021 Mar 9;18:30. doi: 10.1186/s12954-021-00478-4 (PMC7941948; doi:10.1186/s12954-021-00478-4)

**High Concentrations of Illicit Stimulants and Cutting Agents Cause False Positives on Fentanyl Test Strips**

Authors: Tracy-Lynn E. Lockwood*, Alexandra Vervoordt, Marya Lieberman

Table of Contents

[1. Alprazolam, Gabapentin, and Naloxone Buprenorphine Fentanyl Test Strip Reactivity 2](#_Toc51673348)

[2. Fentanyl Test Strips in Cold (4^o^C), Room Temperature (25^o^C), and Warm (40^c^C) Conditions 2](#_Toc51673349)

# Alprazolam, Gabapentin, and Naloxone Buprenorphine Fentanyl Test Strip Reactivity

- Solutions were prepared following the procedure listed in the main text.
- FTS were dipped into solution for 12-15 seconds, set flat to dry, and photographed after 5 minutes following the manufacturer’s instructions.


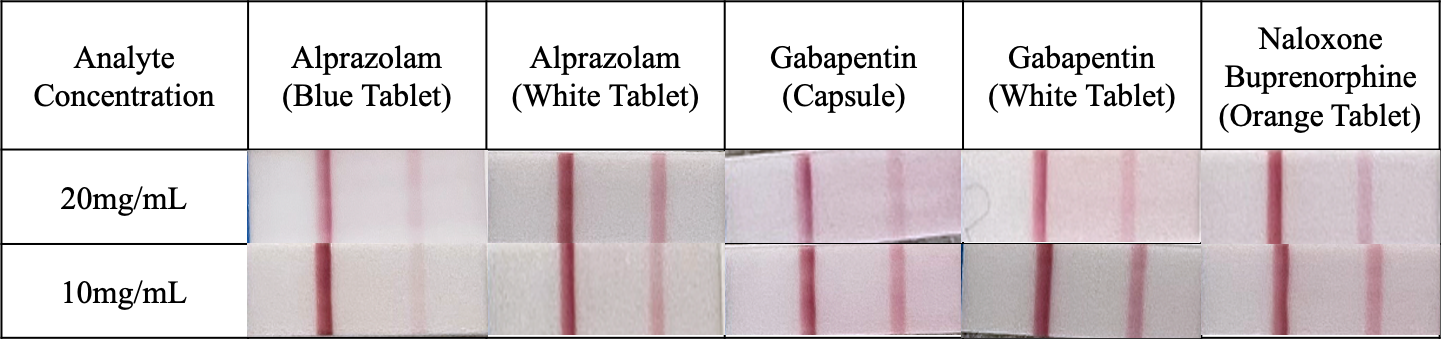


# Fentanyl Test Strips in Cold (4^o^C), Room Temperature (25^o^C), and Warm (40^c^C) Conditions

- Solutions were prepared following the procedure listed in the main text.
- FTS were dipped into solution for 12-15 seconds in the specified temperature condition, set flat to dry, and allowed 5 minutes to process in specific temperature condition.


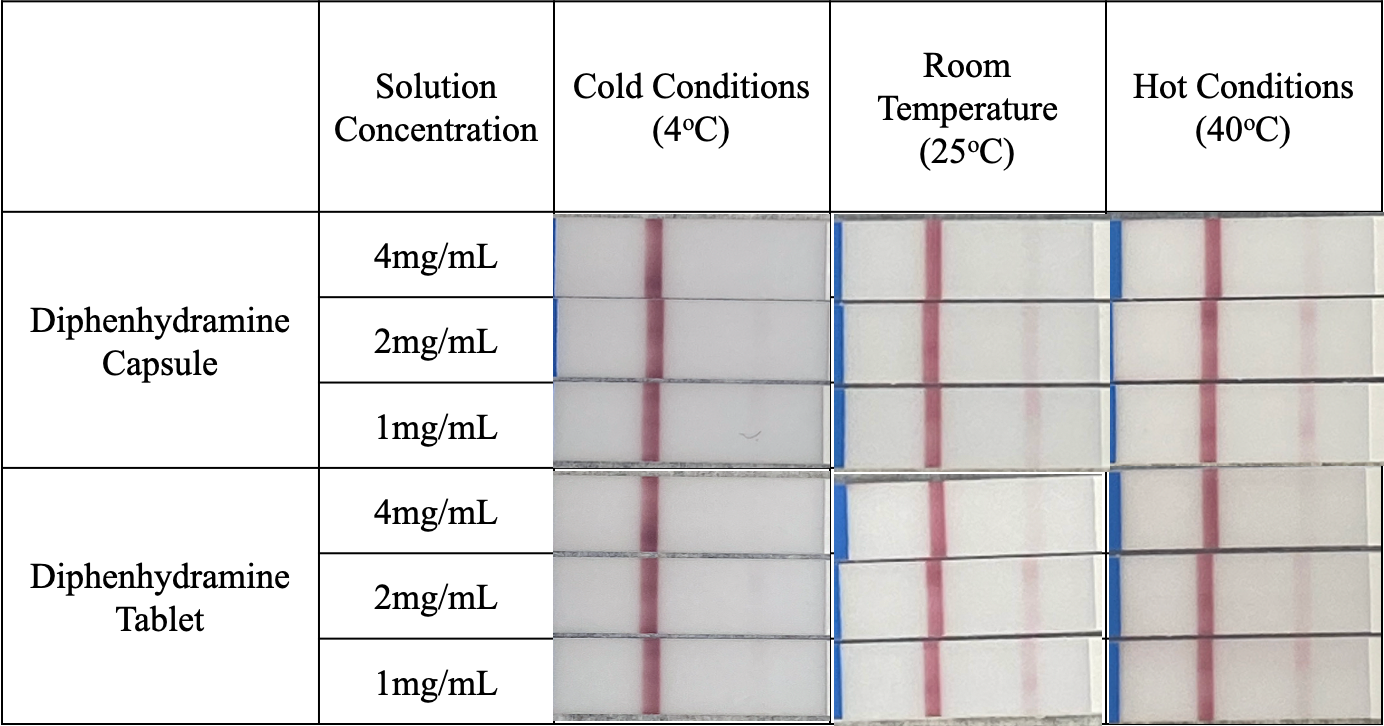

Supplement: Supplementary file 1 — Additional file 1. Supplementary Material. [file 12954_2021_478_MOESM1_ESM.docx]
